# Risk Factors and the Character of Clinical Course of the *Echinococcus multilocularis* Infection in Patients in Poland

**DOI:** 10.3390/pathogens12020199

**Published:** 2023-01-28

**Authors:** Magdalena Stefaniak, Monika Derda, Pawel Zmora, Szymon Pawel Nowak

**Affiliations:** 1 Department of Biology and Medical Parasitology, Institute of Biostructural Basics of Medical Sciences, Poznan University of Medical Sciences, 60-781 Poznan, Poland; 2 Department of Molecular Virology, Institute of Bioorganic Chemistry Polish Academy of Sciences, 61-704 Poznan, Poland; 3 Department and Clinic of Tropical and Parasitic Diseases, Poznan University of Medical Sciences, 60-355 Poznan, Poland

**Keywords:** human alveolar echinococcosis, alveolar echinococcosis, parasitic disease, epidemiology, clinical course, diagnosis

## Abstract

Alveolar echinococcosis (AE) is a chronic zoonotic disease caused by the larval form of *Echinococcus multilocularis*. In humans, it may become a serious chronic infection of the liver which resembles a slow malignant process leading to death when untreated. The aim of the study was an assessment of the risk factors of the *E. multilocularis* infections and the description of AE clinical course in the group of 36 patients with confirmed AE, hospitalized at the Department and Clinic of Tropical and Parasitic Diseases, Poznan University of Medical Sciences between 2013 and 2022. Among the study participants, most patients cultivated land, bred livestock, worked in the forest, or were employed in animal shelters. The *E. multilocularis* infection was diagnosed based on imaging and immunoassay techniques within 6 months in the majority of patients hospitalized in the Department. All patients hospitalized in the Department initiated anti-parasitic therapy at the moment of the diagnosis. Pharmacological treatment combined with surgery was applied in most of the study participants, who were presented with more advanced stages of infection. We conclude the following: 1. For humans in the risk group, regular abdominal imaging examinations and the detection of specific antibodies against *E. multilocularis* are recommended. 2. Regular screening tests in the hyperendemic areas of AE would increase the early detection of the disease and to improve the clinical prognosis in this extremely life-threatening parasitic disease.

## 1. Introduction

Alveolar echinococcosis (AE) is a chronic parasitic infection belonging to zoonotic diseases, where a human is an accidental intermediate host. Due to a recent spread of AE in new geographical regions and the related higher prevalence of the infection among humans, the disease is currently classified in the group of emerging infectious diseases [1]. According to the Polish Regulation of the Minister of the Environment of 29 November 2002 on the classification of pathogens, *Echinococcus multilocularis* is categorized as a microorganism class IV (an equivalent of Biosafety Level 4 pathogens), which cause severe diseases and may pose a serious threat for humans [1].

A strobila of the adult *E. multilocularis* is small, approximately 5 mm in size, has three to five segments (proglottids) and a hooked scolex of 1.1 mm to 2.7 mm in length. The adult gravid proglottids, passed in the feces of the definitive host, contain 200 to 600 eggs which are highly resistant to external biological factors, i.e., they can survive in the humid and low-temperature conditions for approximately one year [2]. In definitive hosts, most commonly carnivores, i.e., foxes, wolves, racoon dogs, lynxes, or coyotes and dogs, an adult *E. multilocularis* resides in the small intestine [2,3]. In the typical life cycle in Poland and other European countries, the definitive host is most frequently the red fox (*Vulpes vulpes*). Further studies showed the common prevalence of *E. multilocularis* in these wild animals in many areas of the northern and south-east parts of Poland [4]. In recent years, the area of distribution of the parasite among definitive hosts and humans has been markedly extended due to a growing population of red foxes, resulting from the prevention program against rabies. The studies on the intensity of *E. multilocularis* dissemination among red foxes in Europe and Japan revealed that 10% of their urban and 50% of their rural population was infected by this parasite [5].

The etiological factor of the disease for a human is the larval form of the *E. multilocularis* cestode, which occupies tissues and the internal organs. A human is an accidental intermediate host and becomes infected by ingesting infective eggs present in food or soil contaminated by the stool of the definitive host [2,3]. Mostly *E. multilocularis* infections were found among the forest workers, farmers, hunters, tanners, forest undergrowth pickers, tourists who visit the endemic areas of *E. multilocularis* residence, and workers of the Veterinary Inspection who collect material to be used in rabies tests in foxes [6]. However, the scale of *E. multilocularis* infection in humans is currently uncertain.

After accidental ingestion, the oncosphere hatch from eggs, penetrate the intestinal wall and are carried by the portal vein to the liver. In 99% of patients, liver is the primary affected organ. Parasite growth is slow, mimicking malignant tumors development [2,3,7]. It is rare that the primary lesions appear in the lungs or the brain of the intermediate host [7]. In the literature, unusual locations of the tapeworm, e.g., the diaphragm, the kidney and/or adrenal gland, and the pancreas, are also reported [8,9]. The reproductive membrane of the *E. multilocularis* infiltrates into the liver parenchyma and expands along the blood vessels and the bile ducts in the infiltrative manner, forming a conglomerate of small vesicles of 0.5–1.2 mm in size. Small cysts form irregular spongy tumor-like lesions with no membrane composed of the connective tissue, so they can grow into the parenchyma of the host’s organ, damaging its function to a great extent. Moreover, they can give metastases into nearby organs, e.g., lymph nodes, diaphragm, and adrenals. At the advanced stage of infection, distant metastases of primary lesions are spread mainly through blood circulation [7]. Therefore, AE is a severe neoplasm-like infection with a poor and always serious clinical prognosis and a risk of death [3].

The aim of this study is to identify the *E. multilocularis* infection risk factors as well as to describe the AE clinical course in patients in Poland.

## 2. Materials and Methods

A multidisciplinary epidemiological and clinical assessment was conducted based on the analysis of a questionnaires (Supplementary Table S1), the findings of radiological data, and the laboratory results of 36 patients aged 17–75 years (mean age 47.9 ± 15.0) with confirmed hepatic AE from various regions of Poland hospitalized at the Department and Clinic of Tropical and Parasitic Diseases, Poznan University of Medical Sciences, between 2013 and 2022.

The *E. multilocularis* infection was diagnosed based on the results of abdominal imaging examinations and ultimately confirmed by the presence of specific IgG antibodies against the Em2–Em18 antigenic complex (Em2plus) in the indirect ELISA immunoenzymatic test (Bordier Affinity Products, Crissier, Switzerland) and by the presence of *E. multilocularis* characteristic 2, 16, and 18 kDa IgG antigens in the reference immunoblotting technique (LDBIO Diagnostics, Lyon, France).

The clinical stage and the dynamics of confirmed AE in humans were determined based on the Parasite–Neighbouring Organs–Metastases (PNM) Classification proposed by the WHO–European Network for Concerted Surveillance of Alveolar Echinococcosis in 2006 [10].

The categorical variables were presented as counts and percentages together with the 95% CI. The 95% CI was calculated using the hybrid Wilson/Brwon method. The statistically significant differences for infection risk factors were calculated with Fisher’s exact test. All statistical analyses were performed with the GraphPad Prism 9 software.

According to the Polish jurisdiction, we did not perform any medical experiment, and therefore the agreement of the Bioethic Committee is not necessary. The patient’s data were collected during routine medical interviews conducted by physicians working at the Department and Clinic of Tropical and Parasitic Diseases. Patients signed standard agreements for non-invasive procedures, including typical physical examinations. The image data, i.e., the ultrasonography scans were taken from medical reports of each patient. All data were anonymized.

## 3. Results

### 3.1. Study Participants Characteristics

**Figure 1:**
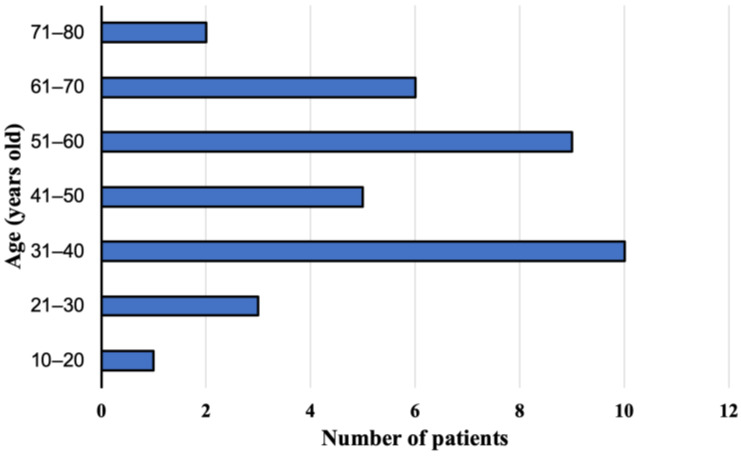
Study participants age at the time of diagnosis, with alveolar echinococcosis from Poland between 2013 and 2022.

The group of 36 patients with the *E. multilocularis* infection confirmed at the Department and Clinic of Tropical and Parasitic Diseases, Poznan University of Medical Sciences, consisted of 21 women (58.3%) aged 17–75 years (mean age 49.6 ± 16.3) and 15 men (41.7%) aged 29–69 years (mean age 45.4 ± 13.2). During the study, 22 (61.1%, 95% CI 43.46–76.86) and 14 (38.9%, 95% CI 23.14–56.54) patients lived in rural and urban areas, respectively. We did not find any significant differences between those two groups. The patients were aged 17–73 years (mean age 46.6 ± 15.0) at the time of diagnosis (Figure 1). Overall, AE was confirmed in 35 (97.3%) adults and one child (2.7%).

### 3.2. Geographic Origin of the AE Patients

**Figure 2:**
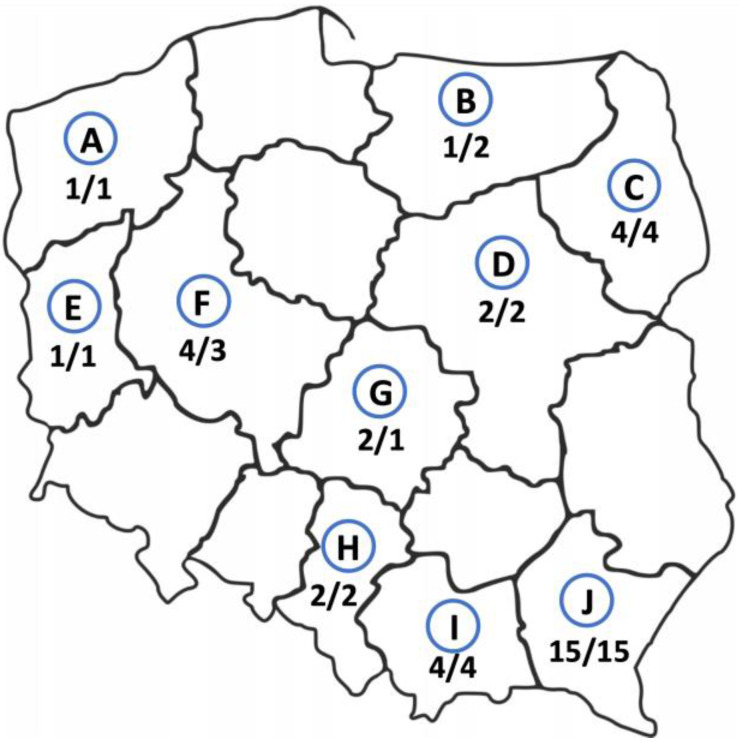
Geographic origin of alveolar echinococcosis patients from Poland between 2013 and 2022. The number of patients infected with *E. multilocularis* currently living in this area/the number of study participants born in this area. **A**—West Pomerania, **B**—Warmian-Masurian region, **C**—Podlaskie region, **D**—Masovia, **E**—Lubuskie region, **F**—Greater Poland, **G**—Łódź region, **H**—Silesia, **I**—Lesser Poland, **J**—Subcarpathian region.

Thirty-four cases of AE were of autochthonous origin, two other *E. multilocularis* cases were imported from other regions of the world, i.e., from Kazakhstan and Lithuania. The study participants came from various regions of Poland, but most of them were from Subcarpathian (n = 15, 42%, 95% CI 25.51–59.24), then from Podlaskie, Lesser Poland, and Greater Poland voivodeships (4 patients from each, 11%, 95% CI 3.11–26.06). In rarer cases, the patients were from Silesian, Łódzkie, and Mazovian regions (2 study participants from each, 6%, 95% CI 0.68–18.66) and from West Pomeranian, Warmian-Masurian, and Lubuskie areas (1 patient from each, 3%, 95% CI 0.07–14.53) (Figure 2). In addition, we found that there are statistically significant differences between the geographic origin of the AE patients.

The places of birth of the infected patients did not substantially differ from their current addresses. The largest group of the patients was born in the Subcarpathian region (n = 15, 42%, 95% CI 25.51–59.24) and still live there. Only three (8.3%, 95% CI 1.75–22.47) patients have changed their places of residence. One person living in the Greater Poland was born in the Warmian-Masurian region, one patient born in Mazovia moved to the Łódź region and one person from Kazakhstan currently lives in the Mazovian region (Figure 2).

### 3.3. E. multilocularis Infection Risk Factors

Among the 36 patients treated in the Department of Tropical and Parasitic Diseases in Poznań, professional contacts with the potential infectious agent were reported by 23 persons (63.9%, 95% CI 46.22–79.18). Most of them worked in agriculture (n = 19, 52.8%, 95% CI 35.49–69.60), two patients claimed having a zootechnical diploma (95% CI 0.68–18.66), one person worked in forestry and one patient was a volunteer in an animal shelter (95% CI 0.07–14.53). The occupations of other subjects (n = 13, 36.1%, 95% CI 20.82–53.78) were not connected with activities increasing the risk of infection. The difference between patients who had professional contact with infectious agents and patients of other occupations was statistically significant with *p*-value 0.0332.

Considering important risk factors for the *E. multilocularis* infection, the study participants mostly declared picking forest fruits (blueberries, blackberries, or mushrooms) (n = 29, 80.6%, 95% CI 63.98–91.81), including 16 women and 13 men. A number of the interviewed patients declared having close contact with dogs (n = 29, 80.6%, 95% CI 63.98–91.81), including 15 women and 14 men. In both cases we observed the statistically significant differences with *p*-values < 0.0001.

In total, 23 individuals (63.9%, 95% CI 46.22–79.18) lived close to the forest, while 24 patients (66.7%, 95% CI 49.03–81.44) reported mowing the grass or wheat near their houses. For both above-mentioned *E. multilocularis* infection risk factors, we observed statistically significant differences with *p*-values of 0.0332 and 0.0090, respectively. Additionally, 15 patients (41.7%, 95% CI 25.51–59.24) drank water directly from natural sources, such as a river or a stream. For 25 (69.4%, 95% CI 51.89–83.65) patients, the main water source in their households was the municipal water, nine (25%, 95% CI 12.12–42.20) study participants used a well, and two (5.6%, 95% CI 0.68–18.66) patients reported both sources being used for food and nutrition purposes. We did not observe significant differences within groups using different main water source.

### 3.4. The Clinical Course of AE

The initial symptoms reported by the patients before the diagnosis of the *E. multilocularis* infection were not characteristic. The patients most frequently suffered from right upper quadrant abdominal discomfort (n = 14, 38.9%, 95% CI 23.14–56.54), jaundice (n = 5, 13.9%, 95% CI 4.67–29.50), loose stools (n = 3, 8.3%, 95% CI 1.75–22.47), and, sometimes, general asthenia, fainting, weight loss, bone pain, a dry cough, itching, or shank oedema (one case for each, 95% CI 0.07–14.53). In this group, three individuals observed more than one symptom (8.3%) and the other seven (19.4%) patients did not mention any clinical symptoms.

**Figure 3:**
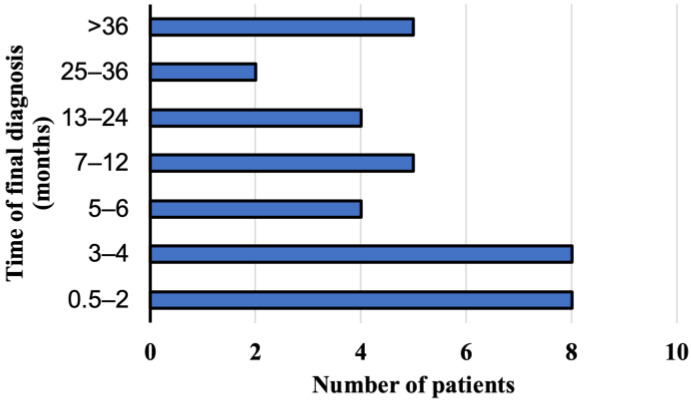
The period of time when the final diagnosis of alveolar echinococcosis has been documented in patients from Poland between 2013 and 2022.

A suspected diagnosis of AE was based on the abdominal ultrasonography in 25 persons (69.4%, 95% CI 51.89–83.65) and on the abdominal computed tomography in eight patients (22.2%, 95% CI 10.12–39.15). Magnetic resonance imaging of the abdomen was the first imaging examination only in three cases (8.3%, 95% CI 1.75–22.47). The imaging analyses were initially performed after the consultations with primary care physicians due to the non-specific discomfort located in the upper quadrant of the abdominal cavity. Patients did not complain of any other symptoms. Ultimate the diagnosis was made by the physicians from the Department and Clinic of Tropical and Parasitic Diseases based on additional imaging tests and immunoassay results. The interval between the initial symptoms and/or the detection of abnormalities in the abdominal imaging investigations was from two weeks to 60 months (mean time 14.2 months). The *E. multilocularis* infection was diagnosed within a year in 25 patients (69.4%, 95% CI 51.89–83.65), between 13 and 24 months in 6 patients (16.7%, 95% CI 6.37–38.21), and over two years in five study participants (13.9%, 95% CI 4.67–29.50). The *E. multilocularis* infection was diagnosed within 6 months in only 20 patients (55.6%, 95% CI 38.10–72.06) hospitalized in the Department (Figure 3).

**Figure 4:**
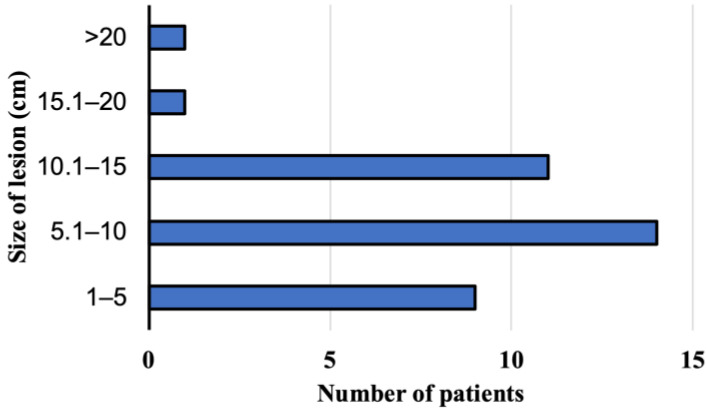
The size of lesion in the liver at the time of diagnosis of alveolar echinococcosis in patients from Poland between 2013 and 2022.

At the time of AE diagnosis at the Department and Clinic of Tropical and Parasitic Diseases, all 36 patients had focal liver lesions. Lesions located peripherally, considering a localization of the hilus and anatomical structures, such as the liver or large bile ducts, were found in 19 patients. The rest of patients had focal lesions, which affected the central part of liver, a hilus, or infiltrate large blood vessels such as liver veins. The sizes of the pathologic lesions detected in the liver were from 1.8 to 21.6 cm (mean size 8.94 cm) at the moment of the diagnosis (Figure 4). Among the study subjects, the parasitic tumors were located primarily in the right liver lobe (n = 23, 64%, 95% CI, 46.22–79.18), the location in both lobes was confirmed in ten patients (28%, 95% CI 14.20–45.19), and three participants (8%, 95% CI 1.75–22.47) presented foci located on the left side of the liver. Advanced pathologic hepatic lesions were identified in the other 17 subjects (47.2%, 95% CI 30.40–64.51) with a central location at the liver hilum; in ten patients (27.8%, 95% CI 14.20–45.19), the infiltration of the surrounding tissues and organs and/or distant metastases to the organs were revealed in 6 patients (16.7%, 95% CI 6.37–32.81), specifically to the lungs (n = 2), to the central nervous system (n = 2), to the lungs and the brain (n = 1), and to the brain and the kidney (n = 1).

### 3.5. The Treatment of E. multilocularis Infection in Patients

In all patients (100%) hospitalized in the Department, anti-parasitic therapy was initiated at the moment of the diagnosis. Pharmacological treatment combined with surgery was applied in most of the study participants who were presented with more advanced stages of infection (n = 27, 75%, 95% CI 57.80–87.88). A group of patients with no affected neighboring organs and with a lack of distant metastases (PNM classification was used), with potentially resectable lesions in the liver according to the CT or MRI imaging, was treated with surgery combined with albendazole treatment after the surgical procedure. Long term treatment with albendazole was reserved for non-operable patients with infiltrations of the liver hilus, vessels such as liver veins and the portal vein, or the presence of metastases to neighboring organs or distant localizations. According to the expert consensus, the patients were treated with 400 mg albendazole twice a day. During the therapy, 26 (72.2%, 95% CI 54.81–85.80) patients did not report any symptoms of treatment intolerance; several individuals suffered from abdominal pain (n = 6, 17.7%, 95% CI 6.37–32.81), diarrhea (n = 2, 5.6%, 95% CI 0.68–18.66), weakness (n = 1, 2.8%, 95% CI 0.07–14.53), or nausea (n = 1, 2.8%, 95%CI 0.07–14.53).

Among 36 patients with the *E. multilocularis* infection, coexisting diseases were diagnosed in 21 persons (58.3%, 95% CI 40.76–74.49), including hypertension (n = 8, 22.2%, 95% CI 10.12–39.15), thyroid diseases (n = 5, 13.9%, 95% CI 4.67–29.50), depression (n = 3, 8.3%, 95% CI 1.75–22.47) and, sometimes, bronchial asthma, anemia, Parkinson disease, and Lyme disease (one case for each, 95% CI 0.07–14.53).

## 4. Discussion

The main reason for AE in humans is considered to be the accidental ingestion of infective eggs present in food or soil contaminated by the stool of the definitive host. Such contamination may result not only from direct contact with feces of definitive hosts but also from touching their fur or soil during gardening or working in the field, from eating unwashed vegetables and fruit, and from drinking contaminated water [6,11,12]. In 2004, Kern et al. reported a list of behaviors that increase the risk of the *E. multilocularis* infection and demonstrated that most of the patients worked in the agricultural sector (70%). An important risk factor was also having a dog hunting for wild animals [11]. Among other risky behaviors, chewing grass blades, professional work in the forest (e.g., cutting down trees), growing plants and herbs in home gardens, as well as the collection and consumption of unwashed strawberries, berries, or other forest fruit were mentioned. According to the authors of the above paper, 80.5% of the patients were the forest undergrowth collectors [11]. In addition, Kern et al. (2004) showed that 65% of the AE subjects lived outside of a town. Our study findings confirm that the occupations associated with agriculture and animal husbandry, as well as forest undergrowth collection, are the main AE risk factors. Surprisingly, we did not observe any statistical differences in the AE patients’ number from rural and urban areas, which may relate to the popularity of outdoor activities, such as blackberry and mushroom collection in the Polish population. On the other hand, no difference between these two areas may be associated with the increasing population of the red fox. According to Vuitton et al., the unexpectedly growing population of urban foxes, climatic changes, and local habits and behaviors of communities in the areas endemically favorable for close contacts with animal reservoirs have an important impact on the rapid spread of AE in Europe and Japan [5]. Further studies confirmed the presence of the endemic spread of the *E. multilocularis* invasion in humans living in areas with a growing population of wild foxes. In the papers by Gawor et al., a significant growth of the prevalence of infection in the population of red foxes was demonstrated based on the studies conducted between 2002 and 2006 compared to the period between 1994 and 1999 [13]. The dominant regions were the Warmian-Masurian region where the growth from 18.8% to 39.6% was observed, the Lesser Poland region with a rise from 0.6% to 21%, the Subcarpathian region with an increase from 7.1% to 36.8%, and the Mazovia region with the increase from 1.3% to 13.5%. A reason for such an expansive growth of the prevalence of *E. multilocularis* is an over 4-fold increase in the population of these animals over 16 years (from nearly 50 thousand in 1990 to 220 thousand in 2006), which is associated with regular rabies elimination actions that have been regularly organized in all regions of Poland since 2002 [14,15,16,17]. This process is inseparable from the growing number of diagnosed AE cases in humans [4]. The above-mentioned growing red foxes population may also be an explanation for the observed statistically significant differences between the number of AE patients from different geographic regions, with the highest number of AE patients from the Sucarpathian region where the red foxes population is also one of the highest observed in Poland [15,17].

An early diagnosis of the *E. multilocularis* infection in humans gives a possibility of an appropriate and optimal treatment to save or significantly prolong the patient’s life and may be associated with a more severe clinical course in elderly patients [18,19]. Unfortunately, in most cases the disease is diagnosed too late, as AE is very rarely taken into account in the differential diagnosis of liver lesions [6].

A diagnosis of AE is limited due to the unspecific clinical presentation, unclear results of imaging examinations, and a high similarity to a malignancy. Additionally, relatively low number of the AE cases lead to the insufficient experience of primary care physicians in the diagnostic process and therapeutic treatment options. When space-occupying lesions in the liver are occasionally detected by imaging techniques, an infection with *E. multilocularis* larval forms is still not too frequently considered. Imaging techniques (ultrasonography, computed tomography scan, and magnetic resonance imaging) revealing an abnormal, irregular mass located in the liver, without a membrane, with necrotic and calcified foci in the center or the periphery, should suggest the presence of this dangerous parasitic disease [20,21]. To confirm the AE, immunodiagnostic tests, such as ELISA and immunoblots, are necessary. The detection of anti-*E. multilocularis* antibodies in the Em2–Em18 ELISA test suggests the AE. However, the ultimate diagnosis must be based on the presence of *E. multilocularis*-specific antigens, i.e., 16 and 18 kDa, in the *Echinococcus* Western blot test [12]. Recently, molecular biology tests have been used often in laboratory diagnostics. Polymerase chain reaction (PCR) with species-specific primers allows for a 100% confirmation of the *E. multilocularis* infection [22].

In our study, the majority of patients were accidentally initially suspected as experiencing a neoplastic process during routine abdominal ultrasonography, before first hospitalization in our Clinic. Since the initial imaging results, the majority of the study participants were ultimately diagnosed with an immunoassay at the Department and Clinic of Tropical and Parasitic Diseases within one year. This relatively short diagnosis time is the result of the extensive knowledge and experience of the Clinic’s employees and the growing awareness of AE among general practitioners and the public, especially in hyperendemic areas. However, it should be highlighted that the average age of analyzed AE patients was relatively high, i.e., 47.9 ± 15.0 years old, which might be associated with a long, asymptomatic clinical course of this zoonosis and an observed small to moderate size of liver lesions. Our observations highlight the importance of imaging-based screening programs, i.e., regular liver ultrasonography. We are also aware that the costs of the screening programs may be very high, therefore it should be limited to the individuals with a high *E. multilocularis* infection risk in the hyperendemic regions, such as the Subcarpathian region. The early AE diagnosis allows for the implementation of the appropriate therapeutic treatment and help patients function properly and maintain their standards of life.

A pathologic lesion observed in the liver may infiltrate the adjacent structures and form metastatic foci to the lungs and/or the central nervous system [23]. A plain chest X-ray may reveal abnormal lesions, suggesting a malignant disease such as focal lesions in the lungs, mediastinal enlargement, or the elevation of the right diaphragm dome. In the presence of metastases, ring-like lesions with calcifications and symptoms secondary to a brain tumor, i.e., oedema of the surrounding tissue, a shift of the structures, and compression signs, may be observed in imaging scans of the head. An ultrasound image of hepatic focal lesions in the course of AE does not determine the type of the implemented therapeutic procedure [20]. The choice of the optimal treatment, determination of the clinical prognosis, and assessment of the therapy effectiveness are based on the PNM classification [10]. It resembles the classification used for the assessment of malignant tumors stages (TNM). In the differential diagnosis of pathologic focal lesions in the liver, non-parasitic hepatic simple cysts, bacterial and amoebic abscesses, tuberculomas, hemangiomas, focal nodular hyperplasia due to hepatocellular cancer, bile duct cancer, metastases to the liver, the *Echinococcus granulosus* infection, and granulomas seen in the course of the invasion of *Toxocara* spp. should be assessed.

In our study group, the pathologic lesions were mainly located in the right lobe of the liver. In many subjects, due to a highly advanced disease, focal lesions were observed in both hepatic lobes. The *E. multilocularis* larva usually migrates to the liver and this is the primary location associated with the parasitic invasion in 99% of patients. The lesions are more frequently located in the right hepatic lobe than in the lungs or the brain of the intermediate host [20]. Due to a still high level of misdiagnoses in differentiation from liver primary and metastatic cancers, each focal lesion of this organ, especially when located around the hilum, should be distinguished from AE [24].

A method of choice in the AE treatment is radical surgery, i.e., a complete surgical resection of the parasitic lesion with a large, 2–3 cm wide safety margin. This procedure frequently involves the removal of two to three liver segments or hemihepatectomy with a concomitant anti-parasitic treatment with albendazole. According to the current recommendations of the expert’s consensus, the radical surgery must be followed by at least a 2-year albendazole chemotherapy treatment and regular clinical, imaging, and serological follow-up over the next 10 years [10]. The surgery is difficult, and its effects depend on the period of infection when the patient is operated on. After the procedure, the patient requires a precise clinical observation to identify early and late complications [25].

In severe inoperable cases and in the advanced stage of the invasion with the infiltration of the adjacent tissues and distant metastases, an alternative method is the administration of the albendazole chemotherapy for the end of the affected patients life [26]. In untreated patients, the mean survival time after the clinical diagnosis is approximately 10 years. Benzimidazoles, i.e., albendazole, used in the therapy of AE since 1975, prolonged the survival time from 6–25% to 80–83% during the 10-year clinical observation [26]. The drugs show exclusively parasitostatic effects against *Echinococcus multilocularis*, so irregular treatment may lead to the relapse of the disease with all of its consequences. Typically, one tablet with 400 mg of albendazole should be taken twice a day by patients. This form of therapy refers to nonoperative patients after the partial resection of the parasitic lesion, following palliative interventions. These patients should undergo regular clinical, imaging, and serological checkups every 3–6 months. In the case of serious complications, such as distant metastases, wasting, bile duct involvement, portal hypertension, or liver failure and cirrhosis, regular clinical and laboratory monitoring should be performed more frequently. The prognosis in AE is always serious and uncertain. The mortality among patients is up to 90%; however, following the surgery combined with a long anti-parasitic chemotherapy, it decreases to 10–14% [2,25,26].

In order to reduce alveolar echinococcosis morbidity, the community should be continuously educated on the serious health consequences of this disease, the serious danger for the humans who live in the endemic regions, and the routes of transmission of this infection in the environment. Health-promoting education should teach hand hygiene and the importance of washing vegetables and forest fruit before consumption, as well as avoiding drinking fresh water from unknown sources. As the parasite *E. multilocularis* eggs die at 60 °C, thermal food processing is recommended. Because the infection may also be transmitted to humans by other definitive hosts, such as domestic dogs, regular anti-parasitic prophylaxis in animals is recommended [27]. In addition, houses should be separated with fences to prevent foxes from entering, wild animals should not be fed, and dumpsters should be secured. Those who are in the AE risk group should wear protective clothing and remember to wash hands frequently [28]. Moreover, imaging screening with ultrasonography of the abdomen should be ensured for the population living in the endemic regions of the infection due to a particular risk of this parasitic disease.

## Supplementary Materials

The following supporting information can be downloaded at: https://www.mdpi.com/article/10.3390/pathogens12020199/s1, Table S1: Epidemiological questionnaire for the patient with alveolar echinococcosis.
