# Supplementary material for: Risk Factors and the Character of Clinical Course of the Echinococcus multilocularis Infection in Patients in Poland
Source: Pathogens. 2023 Jan 28;12(2):199. doi: 10.3390/pathogens12020199 (PMC9962699; doi:10.3390/pathogens12020199)
Supplement: Supplementary file 1 [file pathogens-12-00199-s001.zip › pathogens-2101818-supplementary.pdf]

Supplementary Table S1.

Epidemiological Questionnaire
Alveolar echinococcosis - alveococcosis

1. Name:
2. Year of birth:
3. Sex:
4. Place of birth: voivodship…………….. TOWN/VILLAGE*
5. Current address: voivodship…………………… TOWN/VILLAGE*
6. Profession: farmer, forester, veterinarian, sawmill worker, other ……………………..*
7. House / summer house near the forest: YES/NO *
8. Pets: DOGS , CATS , OTHER: ……………………..*
9. Contact with livestock; work in an animal shelter, in a vet clinic, with wild animals*
10. Eating habits – picking mushrooms, berries, wild strawberries, raspberries in the forest
11. Are fur animals bred near your place of residence? ....
    Which ones?.............
12. Do you mow the grass?......................
13. What source of water do you use in your household? well, municipal water supply *, other ....
14. Have you ever drunk water directly from natural sources (stream, pond, river, lake etc.*) .... When..................
15. The first symptoms: abdominal pain, nausea, vomiting, jaundice, diarrhoea*, other………………………………… (year …………… month………………)
16. Age at the diagnosis:..................
17. When were the first lesions in the liver identified? year …………. month ……….;
    Size of the lesion……………………………………… (cm)
18. Lesions in the liver were detected based on the ultrasound, abdominal computed tomography, abdominal magnetic resonance imaging *
19. Has the blood test for alveococcosis was performed? YES/NO*; year ………..
20. How long was the time from the first symptoms to the final diagnosis?………………………………………
21. Surgeries (please specify the year of the procedure): YES/NO *
    …………………………………………………………………………………
22. Current symptoms:…………………………………………………………….
23. Concomitant diseases:…..……………………………………………..……………
24. Since when was the antiparasitic albendazole (Zentel) administered? …………….
25. Was the albendazole treatment stopped?....................
    Why?.. When?...
    Other received drugs:…………………………………………………………………..
    ………………………………………………………………………………………
